# Suture‐augmented anterior cruciate ligament repair leads to comparable short‐term function but a modestly higher re‐rupture risk than anterior cruciate ligament reconstruction: A systematic review and meta‐analysis

**DOI:** 10.1002/jeo2.70404

**Published:** 2025-09-03

**Authors:** Alessandro Carrozzo, Émilie Bérard, Valerio Nasso, Edoardo Monaco, Jonathan Rioual, Régis Pailhé, Etienne Cavaignac

**Affiliations:** ^1^ Dipartimento di Scienze della Vita, della Salute e delle Professioni Sanitarie Università degli Studi “Link Campus University” Rome Italy; ^2^ Department of Clinical Epidemiology and Public Health, CERPOP INSERM‐University of Toulouse III Toulouse France; ^3^ Department of Orthopaedic Surgery and Traumatology, AOU Sant'Andrea La Sapienza University of Rome Rome Italy; ^4^ Research Methodological Support Unit (USMR), Department of Clinical Epidemiology and Public Health Toulouse University Hospital (CHU) Toulouse France; ^5^ Clinique Aguilera Ramsay Santé Biarritz Biarritz France; ^6^ Department of Orthopaedic Surgery Hôpital Pierre Paul Riquet, CHU de Toulouse Toulouse France

**Keywords:** ACL reconstruction, ACL repair, internal brace, re‐rupture rates, suture augmentation

## Abstract

**Purpose:**

The aim of this study was to conduct a meta‐analysis of the current literature on the treatment of anterior cruciate ligament (ACL) rupture with suture‐augmented ACL repair (SA‐ACLRep) compared to the gold standard ACL reconstruction (ACLR). The meta‐analysis was designed to provide clinical outcomes, including re‐rupture rates (as primary end point), knee stability, functional outcomes, return to sport and complications.

**Methods:**

A systematic literature search was conducted in PubMed, Embase and the Cochrane Library up to 30 August 2024, in accordance with Preferred Reporting Items for Systematic Reviews and Meta‐Analyses guidelines. Comparative clinical studies were included if they conducted a comparative analysis on the clinical outcome of SA‐ACLRep versus ACLR with a minimum of 2 years of follow‐up (FU). The primary outcome was ACL re‐rupture rate; secondary outcomes included complications, knee stability (arthrometer measurements), patient‐reported outcome measures (PROMs) and return‐to‐sport. A random effects model (based on the restricted maximum likelihood method) was used for all pooled analyses.

**Results:**

Four studies met the inclusion criteria and included 687 patients (276 SA‐ACLRep and 411 ACLR). There was no statistically significant difference between the two groups in terms of re‐rupture rates (11.5% with SA‐ACLRep and 8.4% with ACLR; *p* = 0.094). PROMs, including International Knee Documentation Committee, Knee injury and Osteoarthritis Outcome Score subscales, Lysholm, visual analogue scale pain, Single Assessment Numeric Evaluation and Tegner scores, showed no significant differences between SA‐ACLRep and ACLR. No significant differences were found in return to sport rates (72.3% with SA‐ACLRep and 65.0% with ACLR; *p* = 0.541) or timing (mean difference = −0.93 months [95% confidence interval: −2.54, 0.69]; p = 0.261).

**Conclusions:**

SA‐ACLRep with internal bracing and ACLR showed comparable short‐term (≥24 months) FU results, with no statistically significant differences observed in re‐rupture rates, PROMs or return‐to‐play rates. This may suggest that SA‐ACLRep may be a viable alternative for appropriately indicated proximal ACL tears. Heterogeneity in study design, the small number of studies included, the repair timing and reconstruction techniques limit the generalizability of the results.

**Level of Evidence:**

Level III, meta‐analysis of Levels II and III studies.

**Abbreviations**
ACL: anterior cruciate ligament
ACLR: anterior cruciate ligament reconstruction
ADLs: activities of daily living
BPTB: bone‐patellar tendon‐bone
FJS: forgotten joint score
FU: follow‐up
HT: hamstring tendon
IKDC: international knee documentation committee
KOOS: knee injury and osteoarthritis outcome score
LoE: level of evidence
n.r.: not reported
PRISMA: preferred reporting items for systematic reviews and meta‐analyses
PROMs: patient‐reported outcome measures
PROSPERO: international prospective register of systematic reviews
ROBINS‐I: risk of bias in non‐randomized studies of interventions
SANE: single assessment numeric evaluation
SA‐ACLRep: suture‐augmented anterior cruciate ligament repair with internal bracing
SD: standard deviation
VAS: visual analogue scale

## INTRODUCTION

A better understanding of anterior cruciate ligament (ACL) healing, combined with advances in surgical techniques and concerns about the complications associated with ACL reconstruction (ACLR), has led to a recent renewed interest in ACL repair [22]. After rupture, the ACL undergoes a healing response comparable to that of other dense connective tissues. However, unlike extra‐articular ligaments, which typically recover after injury, the intra‐articular ACL develops a synovial layer over the tear site, which can impede repair [26]. Under appropriate conditions, particularly for proximal tears, the ACL has considerable regenerative potential—comparable to that of the medial collateral ligament—due in part to the favourable vascularity of the proximal segment [21, 22, 27]. In the past, primary ACL repair—the first technique historically proposed for ACL rupture—was abandoned because it led to unsatisfactory results in terms of re‐rupture and post‐operative stiffness [5, 8, 10, 20]. Since the abandonment of this technique, around the end of the 1980s, a great deal of research has been conducted on the ACL [26]. Different techniques have been proposed to enhance the healing of the repaired ACL and to protect the delicate healing site of the repaired ACL [15, 16, 29]. These include suture augmentation with a high‐strength suture tape acting as a static stabilizer [23]. The suture augmented technique incorporates this tape into the repair construct, enhancing ligament stability during the healing process functioning as a ‘seatbelt’ to reinforce the native ligament and prevent excessive elongation [23]. This augmentation provides additional mechanical support throughout the healing phase, and recent evidence suggests that suture‐augmented ACL repair (SA‐ACLRep) may support native ligament healing and lead to promising clinical outcomes [28]. However, despite technical advances, ACL repair is still associated with a higher failure rate than autograft reconstruction [2, 11, 28].

Given the renewed clinical interest and the growing body of research, a meta‐analysis is warranted to comprehensively evaluate the evidence, whether SA‐ACLRep provides comparable ACL survival with fewer disadvantages than ACLR. The aim of this study was to conduct a meta‐analysis of the current literature on the treatment of ACL rupture with SA‐ACLRep compared to the gold standard ACLR. The meta‐analysis was designed to provide clinical outcomes, including re‐rupture rates (as primary end point), knee stability, functional outcomes, return to sport and complications. The primary hypothesis was that ACLR may demonstrate an advantage in terms of ACL re‐rupture rates. The secondary hypothesis was that PROMs would be potentially better in the SA‐ACLRep, while potentially providing a faster or equivalent return to sport.

## METHODS

### Literature search

The study was conducted according to the Preferred Reporting Items for Systematic Reviews and Meta‐Analyses (PRISMA) guidelines [24]. The study protocol was registered with the PROSPERO (International Prospective Register of Systematic Reviews) database (registration number: CRD42024573962).

A comprehensive literature search was conducted using PubMed, Embase and the Cochrane Library databases up to 30 August 2024. A comprehensive Boolean strategy combining MeSH terms and free‐text synonyms for (1) ACL, (2) repair or suture augmentation and (3) reconstruction was developed; the full syntax for each database is provided in Appendix S1.

### Inclusion and exclusion criteria

Comparative studies evaluating SA‐ACLRep were included if they met the following criteria: involved patients who underwent SA‐ACLRep and compared with those who underwent ACLR; reported re‐rupture rates and at least one outcome such as complications, return to sport, or PROMs, including International Knee Documentation Committee (IKDC) scores, Knee Injury and Osteoarthritis Outcome Score (KOOS), visual analogue scale (VAS) for pain, Tegner activity scale, Lysholm score or KT‐1000 arthrometer measurements; and had a minimum follow‐up (FU) of 2 years. Studies were excluded if they were non‐clinical (e.g, biomechanical, cadaveric, or animal studies), reviews, case reports, or conference abstracts; clinical articles from the pre‐arthroscopic era; written in a language other than English; involved multiligament injuries (other than the anterolateral ligament); included populations with a history of major ipsilateral knee surgery; had a FU period of less than 2 years; were conducted exclusively in paediatric populations; or did not report re‐rupture rates.

### Study selection and data abstraction

Two independent authors (A.C. and V.N.) independently screened titles and abstracts for eligibility, followed by full‐text assessment of selected articles. Each study was assessed for relevance, and the references of included studies were reviewed to identify additional relevant articles. Disagreements were resolved by discussion with the senior author (E.C.).

Data were extracted independently by two investigators (A.C. and V.N.) using a standardized database.

Before screening began, the author group compiled an a priori list of variables to be extracted. These included: study descriptors required by PRISMA‐P (year, country, design and FU); patient demographics and injury characteristics known to influence repair or graft failure (age, sex, activity level, tear location, Sherman type lesions and tissue quality); surgical‐technique details for subgroup analyses (use of suture augmentation, graft type and fixation); outcomes generally investigated for ACL studies (re‐rupture, complications and patient‐reported outcome measures (PROMs), instrumented laxity and return‐to‐sport). The Tegner activity scale was used to assess the level of sport achieved post‐operatively. Level of evidence was assigned according to the Oxford Centre for Evidence‐Based Medicine [7].

### Risk of bias assessment

The methodological quality of the included studies was assessed by two independent authors (A.C. and V.N.) using the Risk of Bias in Non‐randomized Studies of Interventions (ROBINS‐I) tool [31].

This tool assesses domains such as confounding, selection of participants, classification of interventions, departures from intended interventions, missing data, measurement of outcomes and selection of reported outcomes. Each domain was categorized as having a low, moderate, serious or critical risk of bias. Disagreements between reviewers were resolved by consensus consultation with a third reviewer (E.C.).

### Data analysis

The primary outcome was the rate of re‐rupture after ACL procedures. Secondary outcomes included non‐rupture complications, return to sport, functional outcome scores and knee laxity measures.

Statistical analyses were performed using STATA version 18 (StataCorp LLC), with a significance level set at *p* < 0.05.

To compare the repair and reconstruction groups for qualitative parameters, the study results were tabulated by the number of events and non‐events in each group. When the number of events was zero in at least one cell (event or non‐event) within one group (repair or reconstruction) of a study, a continuity correction value of 0.5 was added to all cells for both groups in that study to estimate the risk ratio. The adjustment is applied during computation; the original data are not modified. We calculate the risk ratio of events for repair versus reconstruction together with its 95% confidence interval (CI).

For continuous outcomes, study results were tabulated with the mean, standard deviation (SD), and total number of subjects in the repair and reconstruction groups. If only the median and range were reported in a study, we estimated the mean and SD using the method described by Hozo et al. [18]. If only the median and interquartile range (IQR) were reported, we estimated the mean and SD using the method described by Wan et al. [34]. We calculated the mean difference with a 95% CI.

We systematically selected a random‐effects model as recommended by Hunter and Schmidt [19]. Studies were then weighted using inverse variance weighting.

Given the small number of included studies, the decision was made to assess publication bias solely through the funnel plot (Appendix S2). Statistical tests, such as Egger's or Begg's, were not conducted, as their reliability is limited in meta‐analyses with few studies. Although no clear evidence of publication bias was detected on visual inspection of the funnel plots, the potential for such bias cannot be excluded, especially considering the limited number of studies and the inherent limitations of visual assessment.

## RESULTS

### Literature search

**Figure 1:**
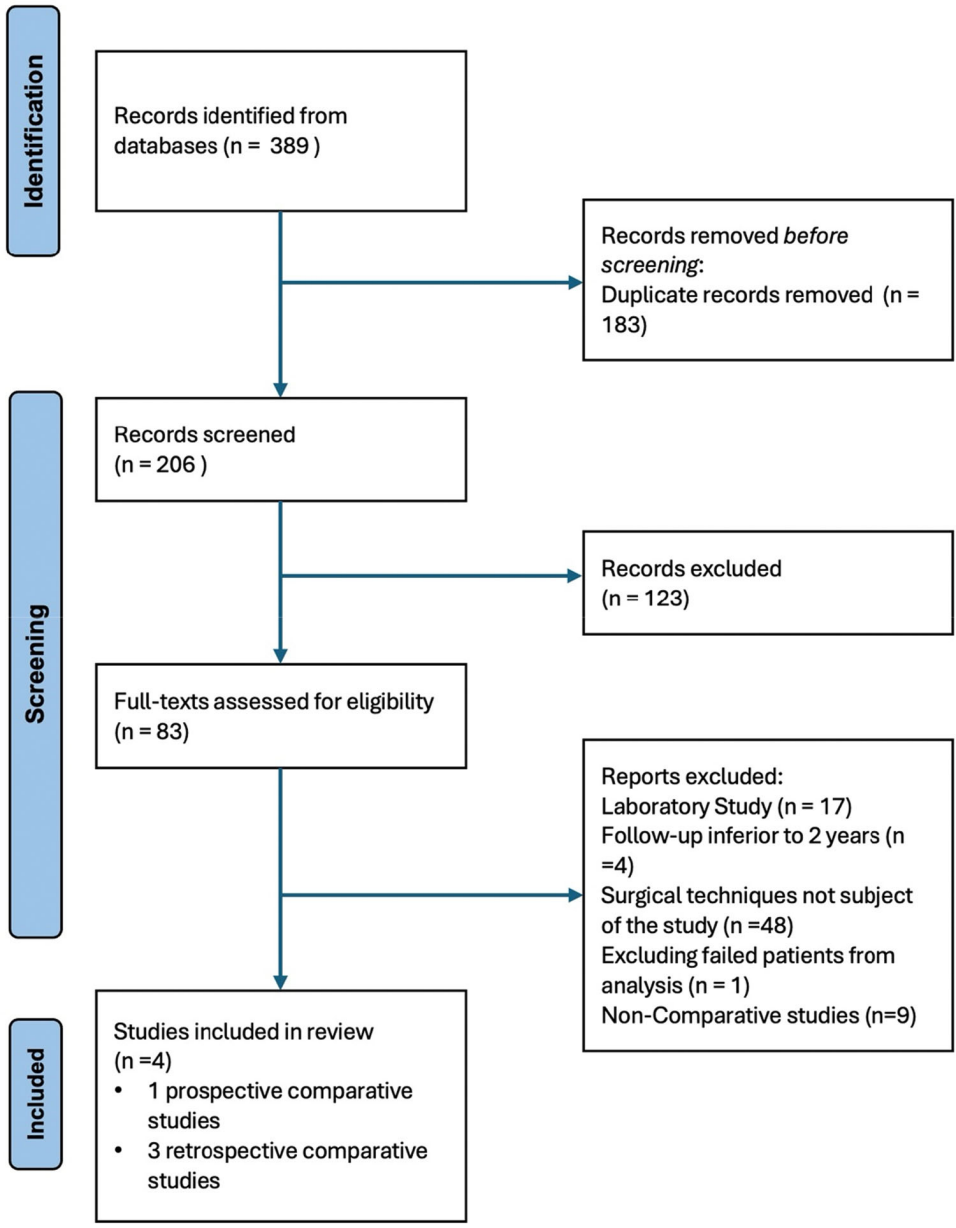
PRISMA (Preferred Reporting Items for Systematic Meta‐Analyses) flow diagram.

A total of 389 records were identified from the database searches. After removing duplicates and applying the inclusion criteria, 83 full‐text articles were assessed for eligibility. Finally, four studies met the criteria and were included in the systematic review (Figure 1).

Included studies varied in design and included nonrandomized prospective and retrospective comparative studies (Table 1).

**Table 1:** Characteristics of included studies with authors, publication years, study design, level of evidence (LoE) [7] and patients' characteristics.

Author year	Study design	LoE	Follow‐up (FU) period in years, mean ± SD	Lost FU, % (SA‐ACLRep/ACLR)	Sample size (SA‐ACLRep/ACLR)	Female, % (SA‐ACLRep/ACLR)	Age, mean ± SD (SA‐ACLRep/ACLR)
Douoguih 2024 [6]	Prospective cohort study	II	Minimum of 2 years	13.3%/10.0%	30/30	56.7%/53.3%	27.5 ± n.r./25.5 ± n.r.
Hopper Wilson 2022 [17]	Retrospective cohort study	III	5.0 ± n.r.	2.2%/0.4%	137/273	44.0%/18.0%	35.0 ± 14.0/28.0 ± 9.0
Ferreira 2022 [11]	Retrospective cohort study	III	2.5 ± 0.4	0.0%/0.0%	75/75	61.3%/64.0%	40.0 ± 11.0/37.6 ± 11.4
Simard 2024 [30]	Retrospective cohort study	III	2.0 ± n.r.	0.0%/3.0%	34/33	51.7%/29.2%	43.2 ± 9.9/35.6 ± 9.8

Abbreviations: ACLR, anterior cruciate ligament reconstruction; n.r., not reported; SA‐ACLRep, suture‐augmented ACL repair; SD, standard deviation.

### Characteristics and quality of the included studies

A total of four studies involving 687 patients were included, with 276 participants in the SA‐ACLRep group and 411 in the ACLR group. Demographic characteristics are reported in Table 1.

Overall, the included studies assessed using the ROBINS‐I tool showed different levels of risk of bias in different domains. Two articles showed low risk in all areas, while two papers showed moderate risk in areas related to selection bias, missing data, and measurement of outcomes (D2, D5 and D6). In Douoguih 2024 [6], there is a moderate risk of bias in the area of missing data (Domain 5) due to unclear reporting of loss to FU in each group and limited information on how incomplete or partial data were handled. In Simard 2024 [30], there is a moderate risk in the domain of selection bias (Domain 2), as the authors excluded patients with ACL rerupture from the final analysis, potentially affecting the comparability of the groups. In addition, there is a moderate risk of outcome measurement (Domain 6) because the KT‐1000 was used without any description of investigator blinding, and patient‐reported outcomes were self‐administered electronically rather than collected by trained healthcare professionals.

**Figure 2:**
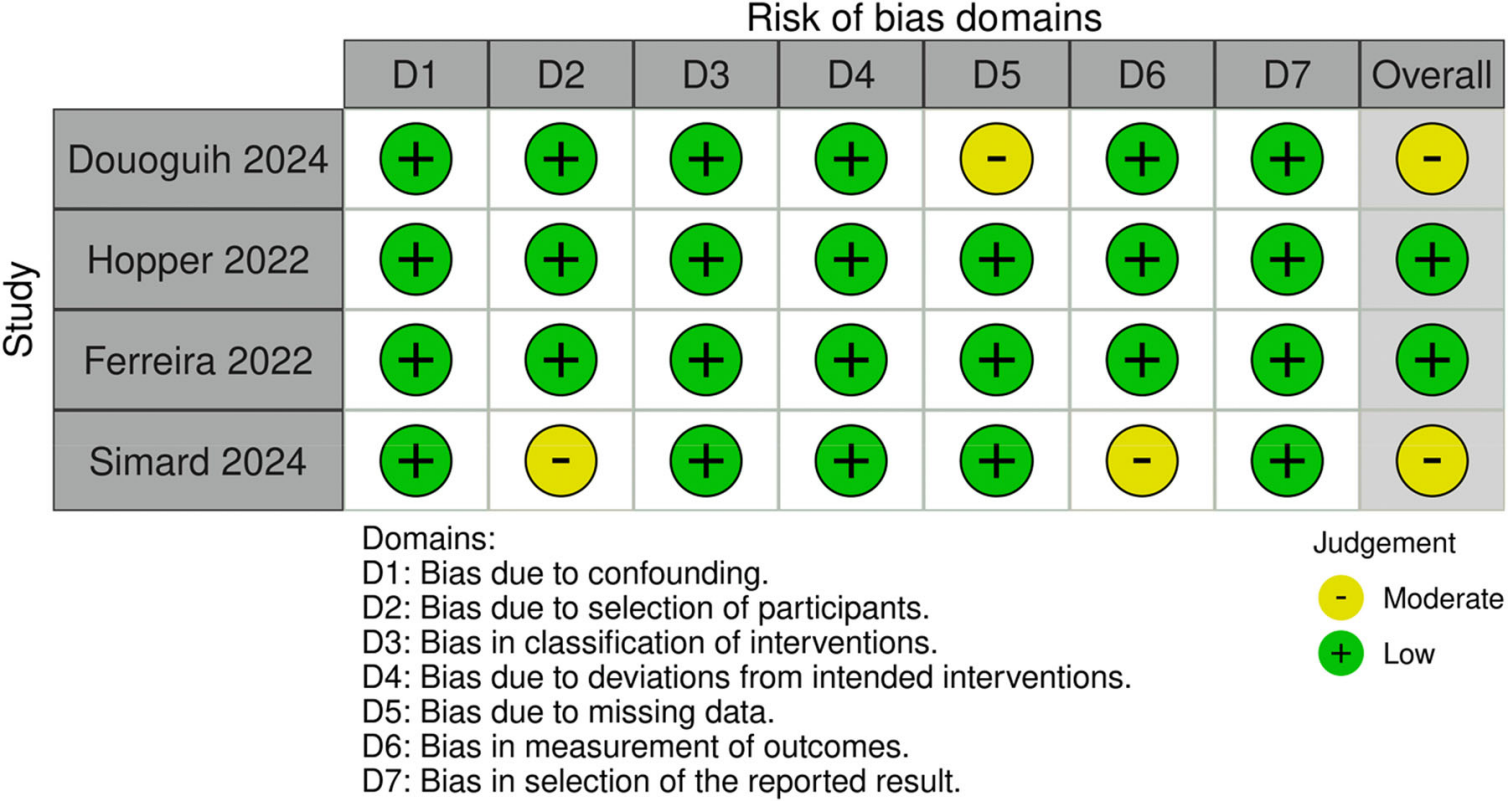
Risk of bias assessment for included studies. This figure provides a summary of the risk of bias across seven domains (D1–D7) for the studies included in the systematic review, evaluated using the ROBINS‐I tool. The risk levels are categorized as low (green), moderate (yellow) or serious (red). The overall risk of bias for each study is displayed in the final column. ROBINS‐I, Risk of Bias in Non‐randomized Studies of Interventions.

Risk of bias assessment is presented in Figure 2.

### Treatment allocation and indications for SA‐ACLRep

Each study defined specific criteria for the inclusion of patients eligible for SA‐ACLRep (Table 2). All the studies indicated an SA‐ACLRep in case of proximal ACL tears, graded according to the Sherman classification. Ferreira et al. required intraoperative confirmation of proximal avulsions with good reducibility.

**Table 2:** Inclusion criteria, timing and reconstruction techniques in comparative studies of primary ACL repair and reconstruction.

Authors	SA‐ACLRep inclusion	Timing criteria	ACLR technique	Failure definition
Douoguih 2024	Femoral avulsion or proximal tear of the ACL (Sherman types I and II)	No	BPTB or soft tissue Quad autograft or soft tissue allograft	ACL reinjury
Hopper Wilson 2022	Acute proximal tears with adequate ACL tissue quality	Within 3 months of injury	Four‐strand semitendinosus‐gracilis graft	ACL reinjury
Ferreira 2023	Intraoperative confirmation of proximal ACL avulsions (Sherman types I and II), tissue quality and reducibility of the remnant.	No	Out‐in BPTB autograft or HT autograft	Revision ACL surgery after re‐rupture
Simard 2024	ACL tear at the femoral attachment with good tissue quality (Sherman type I).	Within 8 weeks of injury	All‐inside ACLR plus suture tape augmentation using hamstring autograft.	Failure/retear after index surgery

Abbreviations: ACL, anterior cruciate ligament; BPTB, bone‐patellar tendon‐bone; HT, hamstring tendon; SA‐ACLRep, suture‐augmented ACL repair.

The timing of repair was specified in some studies, but the results were heterogeneous. For example, Hopper et al. performed repairs within 3 months of injury and Simard et al. limited inclusion to injuries within 8 weeks. Douoguih et al. and Ferreira et al. did not specify timing as a strict inclusion criterion.

Different ACLR techniques were used in the comparison groups. Douoguih et al. used bone‐patellar tendon‐bone (BPTB), quadriceps tendon or soft tissue allografts. Hopper et al. used a four‐strand semitendinosus‐gracilis graft. Ferreira et al. used out‐in BPTB or hamstring tendon (HT) autografts. Simard et al. used an all‐inside ACLR combined with suture augmentation using a hamstring autograft.

### ACL re‐ruptures and reoperations

Overall, 65 out of 676 patients (9.6%) experienced ACL re‐rupture, 31 out of 269 (11.5%) in the SA‐ACLRep group and 34 out of 407 (8.4%) in the ACLR group.

**Figure 3:**
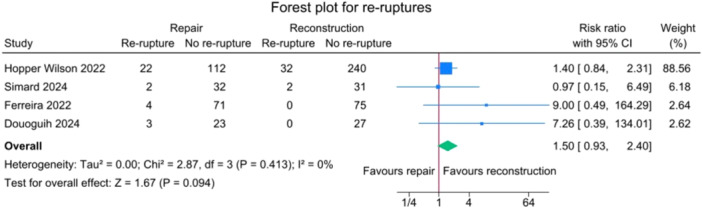
Forest plot comparing re‐rupture rates between SA‐ACLRep and ACLR. ACLR, anterior cruciate ligament reconstruction; CI, confidence interval; SA‐ACLRep, suture‐augmented ACL repair.

There was no statistically significant difference in re‐rupture rates between the repair and reconstruction groups (risk ratio = 1.50 [95% CI: 0.93–2.40]; *p* = 0.094). The studies showed no heterogeneity for this outcome (*I* ^2^ = 0%) (Figure 3).

Surgical re‐intervention not related to ACL rupture was reported in two papers for 32 out of 556 patients, 11 out of 209 in the SA‐ACLRep group and 21 out of 347 in the ACLR group. These are detailed in Table 3.

**Table 3:** Non‐graft‐rupture‐related reinterventions in SA‐ACLRep and ACLR.

Authors	SA‐ACLRep non‐graft rupture‐related reinterventions, *n*/*N* (%)	Description	ACLR non‐graft rupture‐related reinterventions, *n*/*N* (%)	Description
Douoguih et al.	n.r./n.r.	n.r.	n.r./n.r.	n.r.
Hopper et al.	8/134 (6.0%)	3 ipsilateral partial medial meniscectomies; 2 ipsilateral partial lateral meniscectomies; 2 manipulations under anaesthesia and 1 chondroplasty	12/272 (4.4%)	4 ipsilateral partial medial meniscectomies; 1 ipsilateral partial medial and lateral meniscectomy; 3 washouts for infection; 2 chondroplasties; 1 manipulation under anaesthesia and 1 total knee replacement.
Ferreira et al.	3/75 (4.0%)	hardware removal	9/75 (12.0%)	1 secondary meniscectomy; 8 cyclops
Simard et al.	n.r./n.r.	n.r.	n.r./n.r.	n.r.

*Note*: Data are expressed as the number of events over the total number of patients in each group (*n*/*N*), with percentages in parentheses.

Abbreviations: ACLR, anterior cruciate ligament reconstruction; n.r., not reported; SA‐ACLRep, suture‐augmented anterior cruciate ligament repair with internal brace.

There was no significant difference between the repair group and the reconstruction group for the non‐graft rupture‐related reintervention outcome (*p* = 0.646).

**Figure 4:**
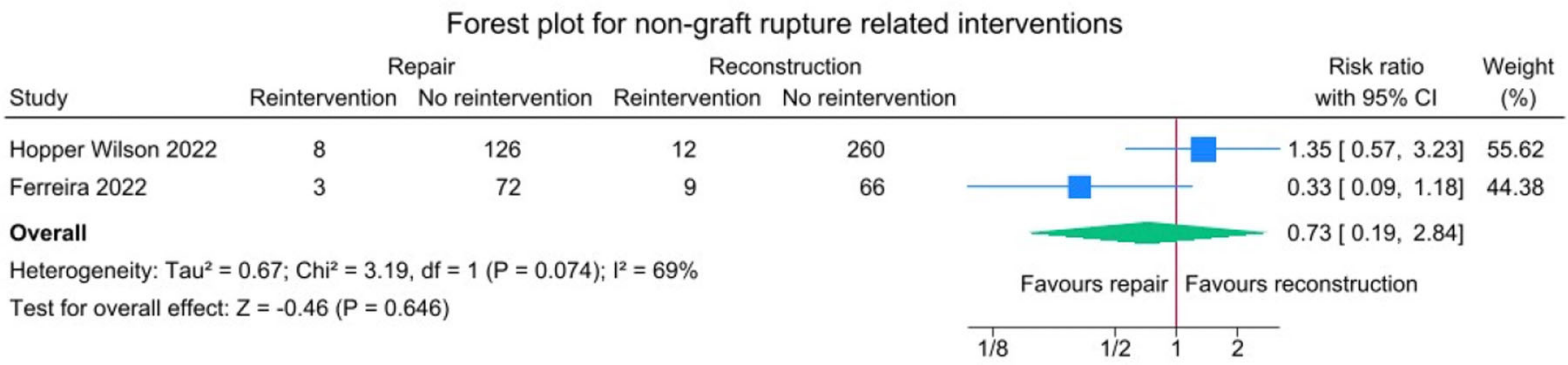
Forest plot comparing non‐graft rupture‐related reintervention rates between SA‐ACLRep and ACLR. ACLR, anterior cruciate ligament reconstruction; CI, confidence interval; SA‐ACLRep, suture‐augmented primary ACL repair.

These studies exhibited high heterogeneity for this outcome measure (*I* ^2^ = 69%) (Figure 4).

### Knee stability outcomes

**Figure 5:**
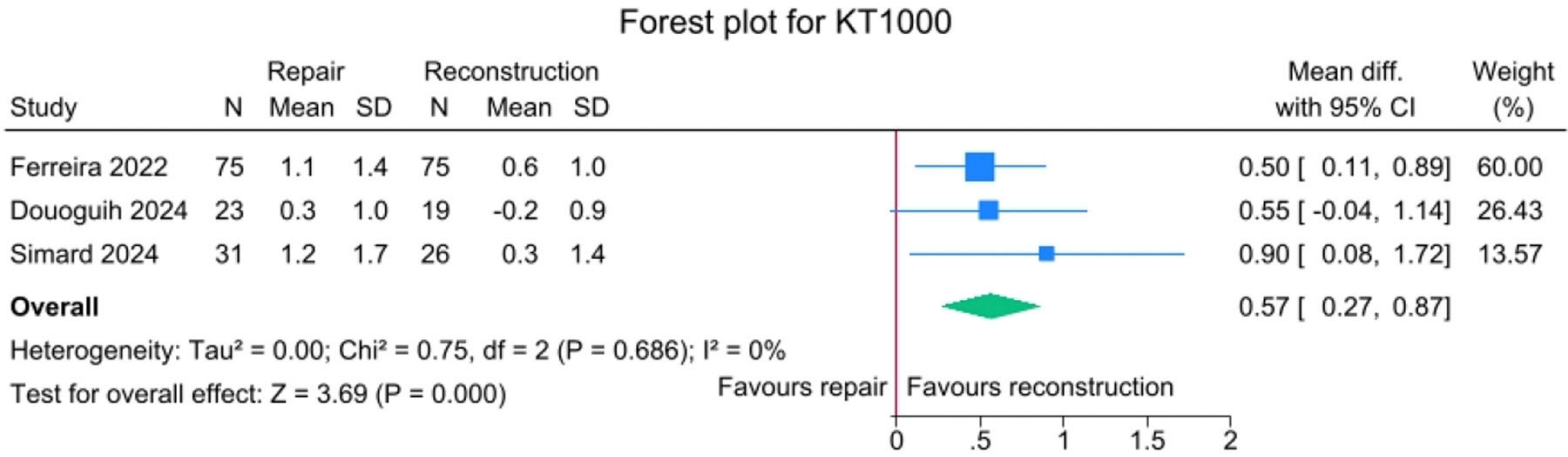
Forest plot of side‐to‐side laximetry, in mm, comparing SA‐ACLRep and ACLR. ACLR, anterior cruciate ligament reconstruction; CI, confidence interval; SA‐ACLRep, suture‐augmented primary ACL repair; SD, standard deviation.

There was a significant difference between the repair group and the reconstruction group for the side‐to‐side laxity, with a mean difference of 0.57 mm [95% CI: 0.27–0.87], favouring the reconstruction group without heterogeneity between studies (*I* ^2^ = 0%) (Figure 5).

### PROMs

**Figure 6:**
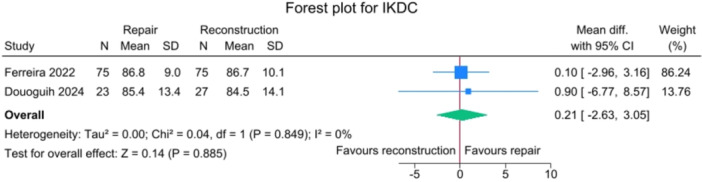
Forest plots showing the distribution of post‐operative IKDC across the studies. CI, confidence interval; IKDC, International Knee Documentation Committee; SD, standard deviation.

**Figure 7:**
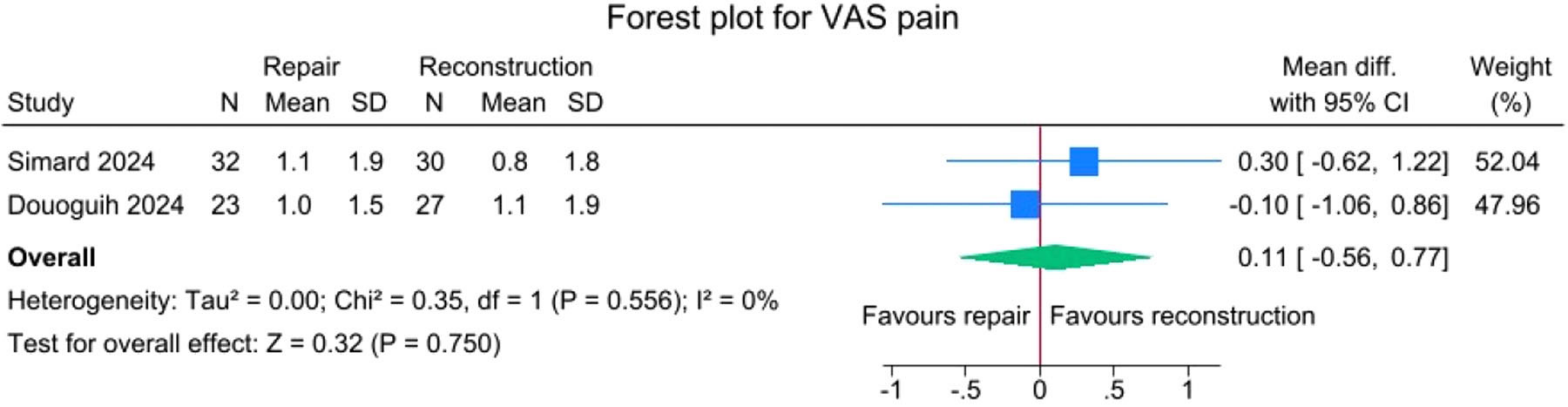
Forest plots showing the distribution of post‐operative VAS pain across the studies. CI, confidence interval; SD, standard deviation; VAS, visual analogue scale.

**Figure 8:**
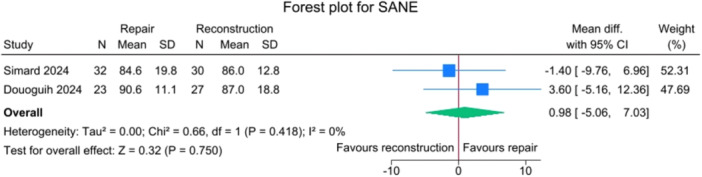
Forest plots showing the distribution of post‐operative SANE across the studies. CI, confidence interval; SANE, Single Assessment Numeric Evaluation; SD, standard deviation.

**Figure 9:**
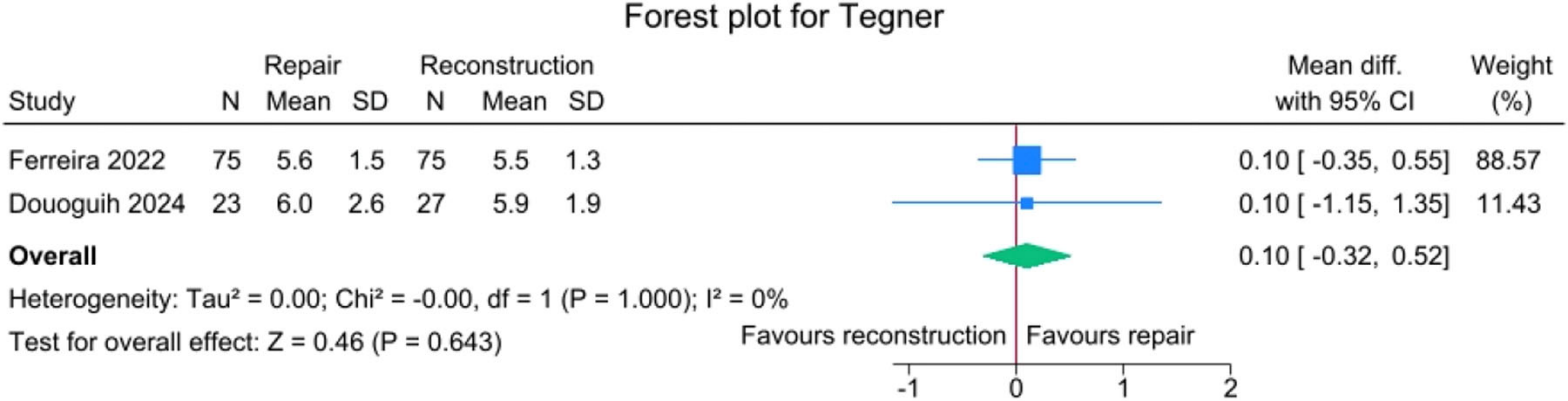
Forest plots showing the distribution of post‐operative Tegner across the studies. CI, confidence interval; SD, standard deviation.

**Figure 10:**
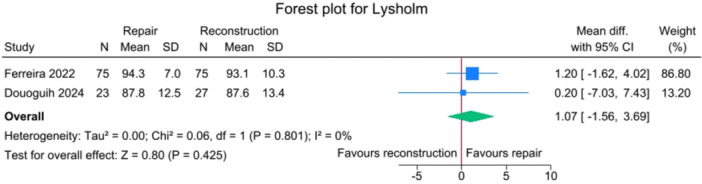
Forest plots showing the distribution of post‐operative Lysholm across the studies. CI, confidence interval; SD, standard deviation.

No significant differences were observed between the repair and reconstruction groups for IKDC scores (*p *= 0.885), VAS pain (*p* = 0.750), Single Assessment Numeric Evaluation (SANE) (*p* = 0.750),Tegner activity scale (*p* = 0.643) and Lysholm score (*p* = 0.425), with no heterogeneity detected (*I* ^2^ = 0%). (Figures 6, 7, 8, 9, 10).

**Figure 11:**
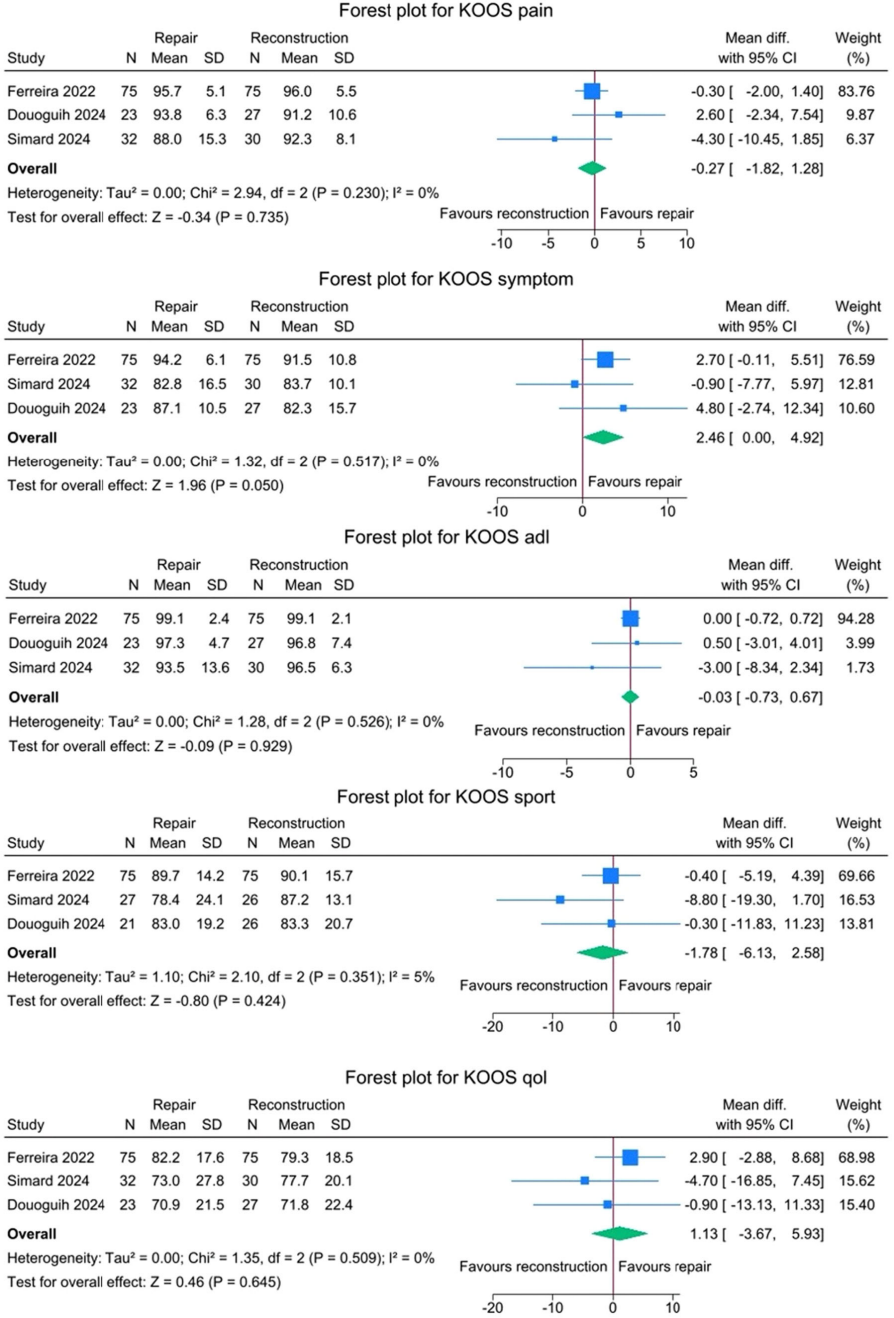
Forest plots comparing KOOS subscales between SA‐ACLRep and ACLR. ACLR, anterior cruciate ligament reconstruction; CI, confidence interval; KOOS, Knee injury and Osteoarthritis Outcome Score; SA‐ACLRep, suture‐augmented primary ACL repair; SD, standard deviation.

Across all KOOS subscales—Pain, Symptoms, activities of daily living (ADLs), Sports and Recreation (Sport) and quality of life—minimal and not statistically significant differences in outcomes between the repair and reconstruction groups were noted, with negligible heterogeneity. Forest plots for KOOS subscales are represented in Figure 11.

### Return to sport

**Figure 12:**
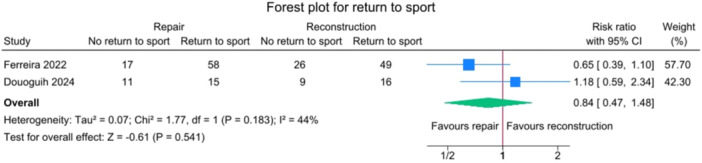
Forest plot comparing return to sport rates between SA‐ACLRep and ACLR. ACLR, anterior cruciate ligament reconstruction; CI, confidence interval; SA‐ACLRep, suture‐augmented primary ACL repair.

**Figure 13:**
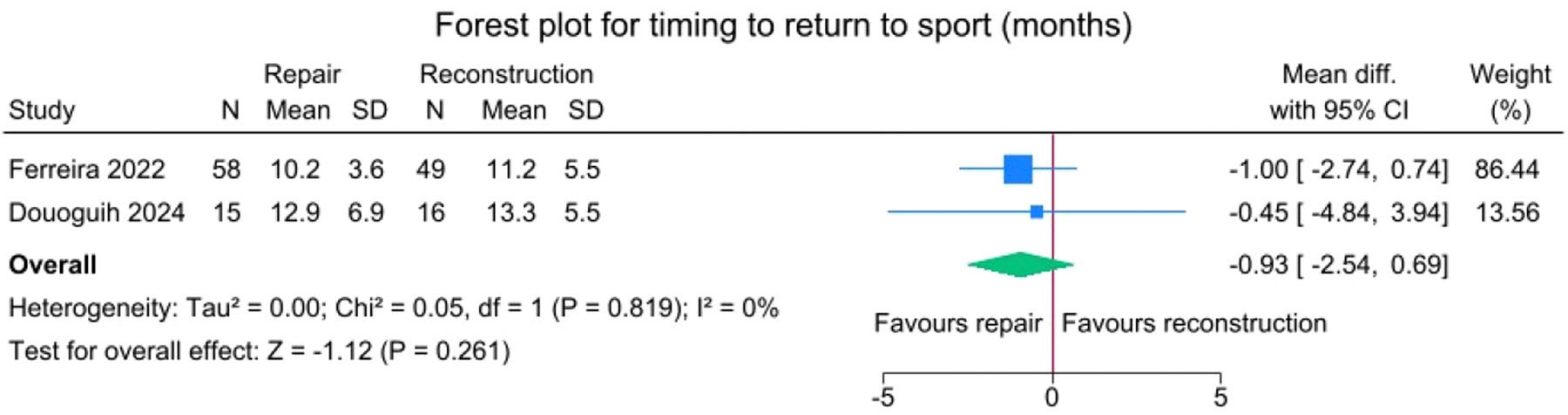
Forest plot comparing timing for return to sport between SA‐ACLRep and ACLR. ACLR, anterior cruciate ligament reconstruction; CI, confidence interval; SA‐ACLRep, suture‐augmented primary ACL repair; SD, standard deviation.

No significant differences were observed between the repair and reconstruction groups in return‐to‐sport rates (*p* = 0.541) or in the timing of return to sport (*p* = 0.261) (Figures 12 and 13).

## DISCUSSION

The main findings of this study are that SA‐ACLRep provides no statistically significant difference compared to ACLR in terms of re‐rupture rates, patient‐reported outcomes and return to sport.

The analysis showed no statistically significant difference in re‐rupture rates between SA‐ACLRep and ACLR (*p* = 0.094).

Historically, primary ACL repair was largely abandoned because high re‐rupture rates and post‐operative stiffness produced unsatisfactory outcomes [5, 8, 10, 20]. Since then, improvements in surgical technology, refined operative techniques, and modern rehabilitation protocols have markedly enhanced primary ACL repair results, and SA‐ACLRep appears to benefit from the added stabilizing support of the augmentation during the vulnerable healing process. This ligament augmentation technique aims to reinforce the ligament, while allowing early mobilization [23]. Still, in the current paper, no direct comparisons have been conducted between ACL repair with and without suture augmentation, making it impossible to determine the specific beneficial effects of suture augmentation [29].

Different trials showed, in some aspects, comparable outcomes between ACL repair and reconstruction [12, 14, 25]. However, regarding ACL re‐ruptures, the majority demonstrated a trend favouring ACLR, as observed in this study. Ferretti et al. reported the results of a prospective nonrandomized comparative study involving 53 patients receiving ACL Repair and 47 patients receiving ACLR [13]. In this cohort, the authors found no significant differences between groups with respect to ipsilateral second ACL injury rates (ACL Repair group 3.8% vs. ACLR group 2.1%; *p* = 0.63). Fayard et al. conducted a retrospective cohort study comparing patients who underwent ACL repair reinforced with a ‘biological internal brace’ using a gracilis autograft to those who underwent standard ACLR [9]. They matched 49 couples of patients and assessed the clinical outcomes at a minimum 2 years FU. The authors found a trend for a higher failure rate in the ACL Repair group, not reaching statistical significance (6.1% [3/49] vs. 0%; *p* = 0.08).

Knee stability, measured by side‐to‐side laxity with instrumented arthrometers, significantly favoured ACLR, with a mean difference of 0.57 mm (95% CI: 0.27–0.87; p < 0.001). However, it is important to consider the sensitivity of the instruments, which are not always accurate to within half a millimetre. In addition, a half millimetre difference in anterior translation between the two knees is unlikely to be clinically relevant.

One of the advantages of ACL primary repair is its potential for improved PROMs, probably due to a less invasive procedure and to the maintenance of the native ACL tissue. In the current study, no significant differences were observed between SA‐ACLREP and ACLR for PROMs, including IKDC, VAS pain, SANE, Tegner activity scale and KOOS subscales. However, SA‐ACLREP showed a tendency towards a better KOOS Symptoms score (*p* = 0.05). The fact that no differences in PROMs were found in the current meta‐analysis may be due to several factors. First, the data were pooled from relatively small trials, and thus, the studies, even if merged, can be underpowered to find differences in terms of PROMs between the two groups. Second, with regard to surgical techniques, SA‐ACLRep was performed with slightly different techniques in the included trials, and the ACLR arms used different grafts, which may mask differences in outcomes. Some centres only performed SA‐ACLRep within an early window, while others did not specify timing as an inclusion criterion, and early versus delayed repair may result in different functional outcomes. In addition, many PROMs, such as the included IKDC and KOOS, may have a ceiling effect, making it more difficult to detect small differences. Also, even if the SA‐ACLRep group can show a faster early recovery, by the time patients reach 2–3 years post‐operatively of the included studies, the difference in PROMs may be smaller. Then, importantly, traditional scores such as IKDC, KOOS and Tegner are validated, but they may not capture subtle proprioceptive benefits or the patient's subjective sense of ‘normalcy’. In contrast, the Forgotten Joint Score (FJS) may be more sensitive to these differences. Notably, several studies have reported that patients undergoing ACL repair achieve higher scores on the FJS, which emphasizes the patient's ability to ‘forget’ the operated joint, indicating better joint perception and integration during daily activities [2, 11, 12, 32, 33]. Vermeijden et al. conducted a study evaluating subjective preference and functional outcomes of patients receiving primary ACL repair in one knee and ACLR in the contralateral side [33]. They included 21 patients and found that 33% preferred the repaired knee, 11% preferred the reconstructed knee, and 56% had no preference. However, 78% reported that their repaired knee was less painful during rehabilitation, and 83% reported an earlier return of range of motion after repair.

Return‐to‐sport rates and timing showed no significant differences between the ACL repair and ACLR groups (*p* = 0.541 and *p* = 0.261, respectively). Return to sport is multifactorial. External factors such as team or season schedules, access to rehab facilities, or personal choice (not to return to competitive sports) also influence return‐to‐play rates more than which surgical procedure was performed. Also, age was higher in the SA‐ACLRep population. Younger people may be more active and motivated to undergo intensive rehabilitation and return to sport. However, various studies examining post‐operative rehabilitation and return to sports have demonstrated that patients undergoing ACL repair outperform those undergoing ACLR in isokinetic strength tests at 6 months post‐operatively [9, 11]. This advantage persists, with superior performance observed even at 2 years following surgery [1]. This can be linked to a better early ability to return to sports at six months, with respect to standard ACLR [12]. However, when investigating athletic performance after ACL Repair or ACLR, Fayard et al. found that the Santy Athletic Return to Sport test score was significantly higher in the ACLR group (69.7 ± 16.6% [range, 19%–100%] vs 61 ± 16.8% [range, 19%–100%]; *p* = 0.001) [4, 9].

A very recent meta‐analysis by Conde et al. pooled nine cohorts (1049 patients) and reported a significantly higher failure rate after suture tape‐augmented ACL repair (risk ratio [RR]: 3.62) together with similar PROMs, better hamstrings strength and slightly shorter operating time when compared with ACLR [3]. Our findings are consistent in that functional outcomes and return‐to‐sport timing did not differ; however, in our more stringent research, the increase in re‐rupture risk did not reach statistical significance (RR 1.88; *p* = 0.094). That result in the Conde et al. paper was driven predominantly by a single paediatric cohort (Gagliardi 2019, age of the included patients 7–18 years), whereas two additional studies with very short FU (<18 months) contributed no re‐ruptures and widened heterogeneity. In contrast, our review limited inclusion to adult or skeletally mature patients, required ≥24 months' mean FU, and mandated concurrent reconstruction comparators. These stricter criteria reduced heterogeneity to 0% and led to a non‐significant increase in failure risk (RR: 1.88; *p* = 0.094). Both reviews suggest that suture‐augmented repair provides functional results comparable to reconstruction, but that failure risk may be higher in younger patients.

This study has several limitations. First, the number of included studies was small (and the meta‐analysis is likely underpowered to detect any significant differences). Moreover, these studies had varying levels of methodological rigour, with some having a moderate risk of bias in key areas such as outcome measurement and reporting. In addition, the heterogeneity of surgical techniques, inclusion criteria, including timing criteria, and FU between studies limits the generalizability of the findings. Designs of the included studies varied (all were non‐randomized, with retrospective or prospective comparative cohorts), and the lack of randomization introduces potential confounding bias, affecting the comparability of repair and reconstruction groups. Also, interpretation of PROMs is limited, as only the KOOS was reported in three or more studies, while all other PROMs appeared in at most two cohorts. However, we tried to mitigate these limits using a rigorous methodology, including adherence to PRISMA guidelines, a comprehensive literature search, and assessment of bias using the ROBINS‐I tool. Nevertheless, a 9‐month interval elapsed between the search cut‐off date and manuscript submission, due to the time needed for data extraction, analysis, manuscript drafting and iterative co‐author revisions, which could be a limitation.

## CONCLUSION

SA‐ACLRep with suture augmentation and ACLR showed comparable short‐term (≥24 months) FU results, with no statistically significant differences observed in re‐rupture rates, PROMs or return‐to‐play rates.

This may suggest that SA‐ACLRep may be a viable alternative for appropriately indicated proximal ACL tears. However, heterogeneity in study design, the small number of studies included, the repair timing, and reconstruction techniques limit the generalizability of the results.

## AUTHOR CONTRIBUTIONS

All authors contributed to the study. Etienne Cavaignac and Edoardo Monaco have ideated the study and established the study design. Material preparation, data collection and analysis were performed by Valerio Nasso, Alessandro Carrozzo, Jonathan Rioual and Émilie Bérard. The first draft of the manuscript was written by Alessandro Carrozzo, and Etienne Cavaignac, Edoardo Monaco and Régis Pailhé had substantially edited the draft. All authors read and approved the final manuscript.

## CONFLICT OF INTEREST STATEMENT

Etienne Cavaignac: Consultant for Arthrex, Amplitude and Biobank. Edoardo Monaco: Consultant for Arthrex. The remaining authors declare no conflicts of interest.

## ETHICS STATEMENT

The ethics statement is not available.

## Supporting information

Appendix S1.

Appendix S2.

## Data Availability

The data sets generated and analyzed during the current study are not publicly available but are available from the corresponding author on reasonable request.
