# Supplementary material for: Suture‐augmented anterior cruciate ligament repair leads to comparable short‐term function but a modestly higher re‐rupture risk than anterior cruciate ligament reconstruction: A systematic review and meta‐analysis
Source: J Exp Orthop. 2025 Sep 3;12(3):e70404. doi: 10.1002/jeo2.70404 (PMC12406634; doi:10.1002/jeo2.70404)
Supplement: Supplementary file 1 — Appendix S1. [file JEO2-12-e70404-s001.docx]

Appendix_1

PubMed:

(("Anterior Cruciate Ligament"[Majr] OR "anterior cruciate ligament"[tiab] OR ACL[tiab])

AND

(("primary repair"[tiab] OR "direct repair"[tiab] OR "ACL repair"[tiab])

AND

("suture augmentation"[tiab] OR "suture-augmented"[tiab]

OR "internal brace"[tiab] OR "internal bracing"[tiab]))

AND

("ACL reconstruction"[tiab] OR reconstruct*[tiab])

AND

(Comparative Study[pt] OR randomized[tiab] OR cohort[tiab] OR matched[tiab]))

NOT

(revision[tiab] OR posterior[tiab])

AND humans[mh] AND english[lang]

AND "2000/01/01"[PDAT] : "2024/08/30"[PDAT]

Embase:

('anterior cruciate ligament'/mj OR 'anterior cruciate ligament':ti,ab OR acl:ti,ab)

AND

(('primary repair':ti,ab OR 'direct repair':ti,ab OR 'acl repair':ti,ab)

AND

('suture augmentation':ti,ab OR 'suture-augmented':ti,ab

OR 'internal brace':ti,ab OR 'internal bracing':ti,ab))

AND

('acl reconstruction':ti,ab OR reconstruct*:ti,ab)

AND

(comparative study/ or cohort*:ti,ab or match*:ti,ab or random*:ti,ab)

NOT

(revision*:ti,ab or posterior*:ti,ab)

AND [english]/lim AND [human]/lim AND [2000-2024]/py

Cochrane:

(("Anterior Cruciate Ligament" OR ACL):ti,ab,kw)

AND

(("primary repair" OR "direct repair" OR "ACL repair"):ti,ab,kw

AND

("suture augmentation" OR "internal brace" OR "internal bracing"):ti,ab,kw)

AND

("ACL reconstruction" OR reconstruct*):ti,ab,kw

AND

(cohort OR comparative OR randomized OR controlled):ti,ab,kw
