# Supplementary material for: Suture‐augmented anterior cruciate ligament repair leads to comparable short‐term function but a modestly higher re‐rupture risk than anterior cruciate ligament reconstruction: A systematic review and meta‐analysis
Source: J Exp Orthop. 2025 Sep 3;12(3):e70404. doi: 10.1002/jeo2.70404 (PMC12406634; doi:10.1002/jeo2.70404)
Supplement: Supplementary file 2 — Appendix S2. [file JEO2-12-e70404-s002.docx]

Appendix 2: Forest plots and corresponding funnel plots for all study outcomes

**Comparison of qualitative parameters in repair group vs reconstruction group**

**Re-ruptures**

***
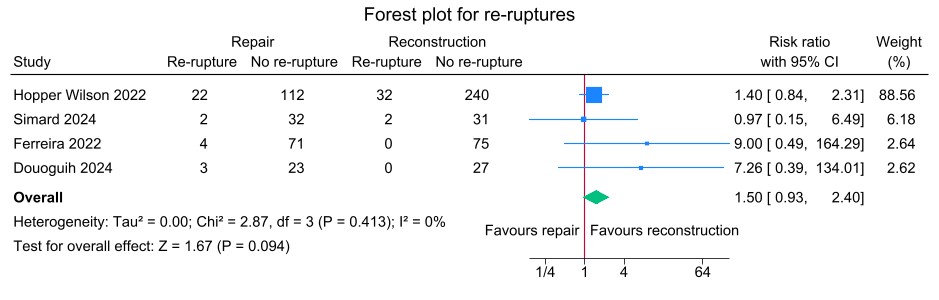
***

***Forestplot_MA_rerupture.pdf***

There is no significant difference between the repair group and the reconstruction group for the re-rupture outcome. A trend is observed favouring the reconstruction group (p=0.095).

These studies exhibit none heterogeneity for this outcome measure (I²=0%).

**
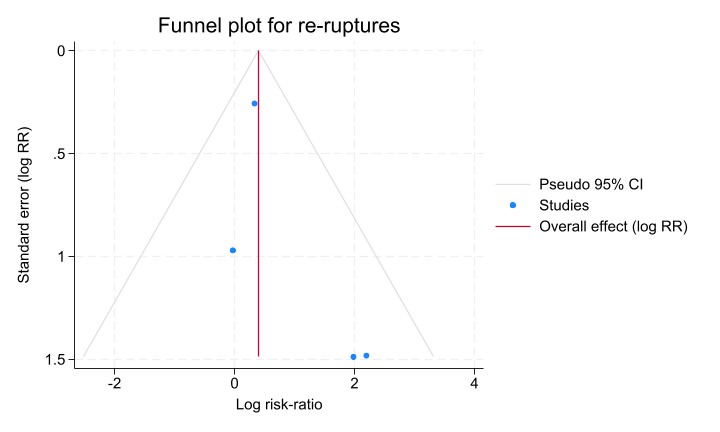
*Funnelplot_MA_rerupture.pdf***

The funnel plot for the re-rupture outcome does not reveal any evidence of publication bias.

**Non-graft rupture related reinterventions**


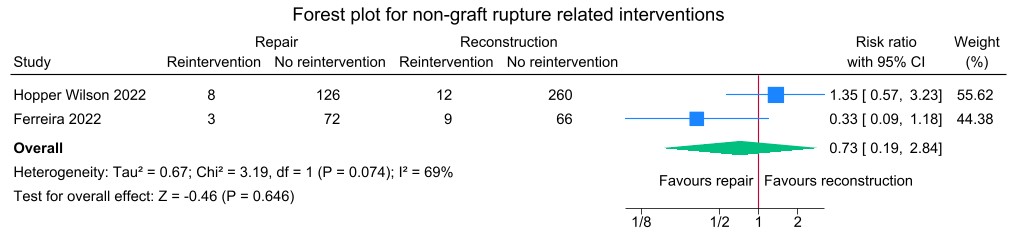


***Forestplot_MA_non_gr_rupt_rel_re.pdf***

There is no significant difference between the repair group and the reconstruction group for the non-graft rupture related reinterventions outcome (p=0.646).

These studies exhibit high heterogeneity for this outcome measure (I²=69%).

**
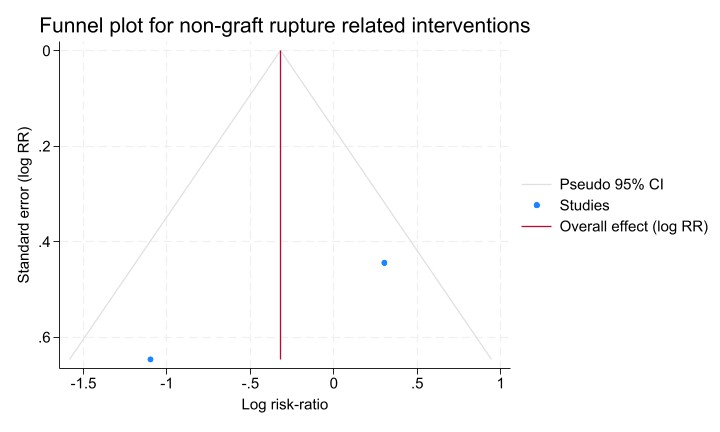
**

***Funnelplot_MA_non_gr_rupt_rel_re.pdf***

The funnel plot for non-graft rupture related reinterventions outcome does not reveal any evidence of publication bias.

**Return to sport**


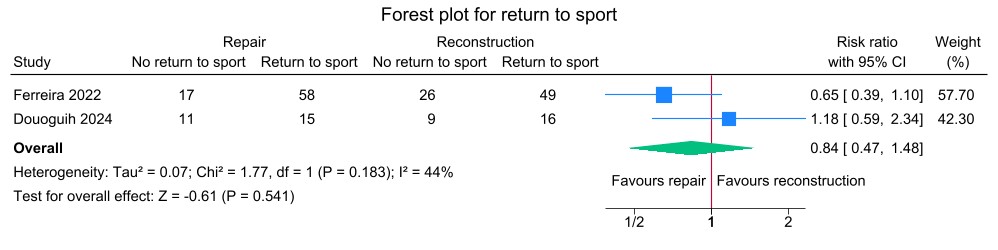


***Forestplot_MA_return_sport.pdf***

There is no significant difference between the repair group and the reconstruction group for the return to sport outcome (p=0.541).

These studies exhibit moderate heterogeneity for this outcome measure (I²=44%).

**
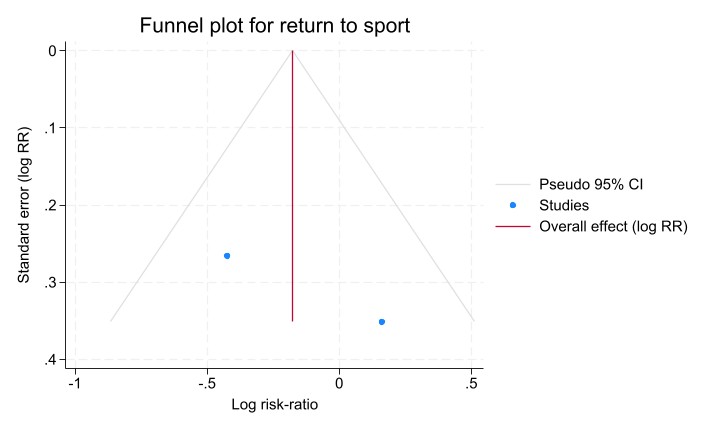
**

***Funnelplot_MA_return_sport.pdf***

The funnel plot for return to sport outcome does not reveal any evidence of publication bias.

**Comparison of quantitative parameters in repair group vs reconstruction group**

**Timing to return to sport (months)**


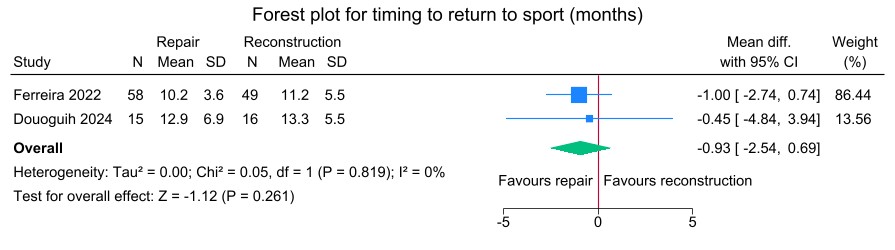


***Forestplot_MA_timing_return_sport.pdf***

There is no significant difference between the repair group and the reconstruction group for the timing to return to sport outcome (p=0.261).

These studies exhibit none heterogeneity for this outcome measure (I²=0%).

**
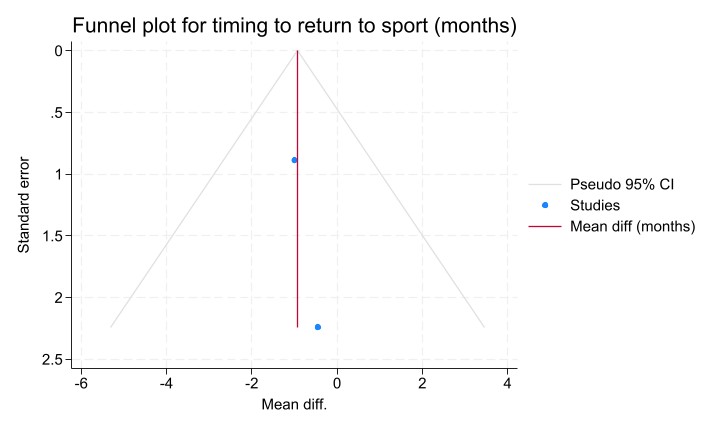
**

***Funnelplot_MA_timing_return_sport.pdf***

The funnel plot for the re-rupture outcome does not reveal any evidence of publication bias.

**IKDC**


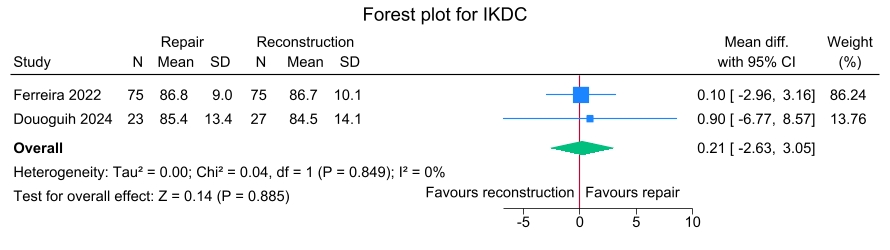


***Forestplot_MA_IKDC.pdf***

There is no significant difference between the repair group and the reconstruction group for the IKDC outcome (p=0.885).

These studies exhibit none heterogeneity for this outcome measure (I²=0%).

**
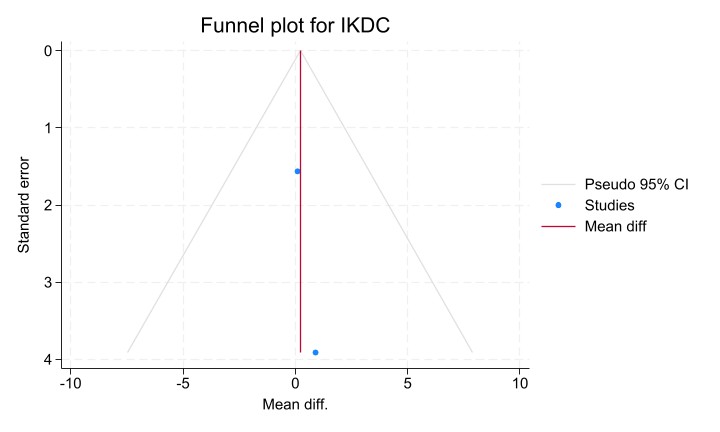
**

The funnel plot for the IKDC outcome does not reveal any evidence of publication bias.

**KOOS pain**


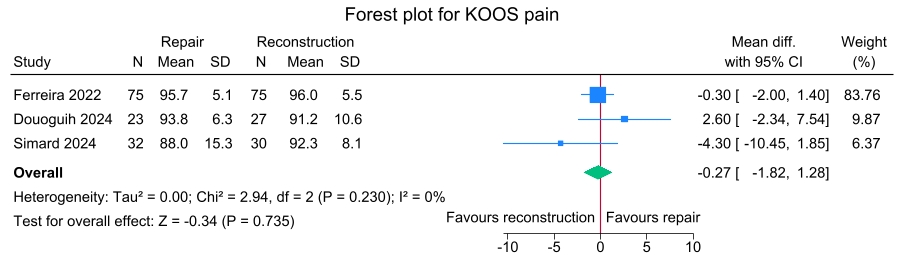


***Forestplot_MA_KOOS_pain.pdf***

There is no significant difference between the repair group and the reconstruction group for the KOOS pain outcome (p=0.735).

These studies exhibit none heterogeneity for this outcome measure (I²=0%).

**
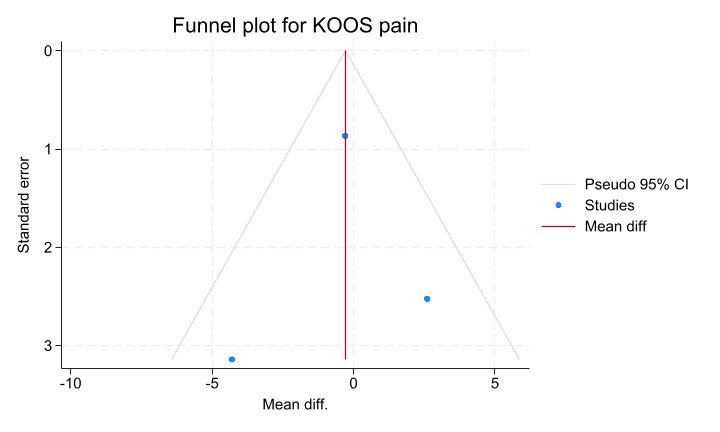
**

***Funnelplot_MA_KOOS_pain.pdf***

The funnel plot for the KOOS pain outcome does not reveal any evidence of publication bias.

**KOOS symptom**


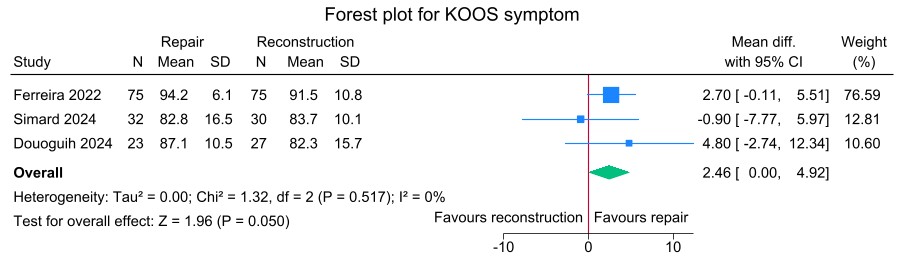


***Forestplot_MA_KOOS_symptom.pdf***

There is no significant difference between the repair group and the reconstruction group for the KOOS symptom outcome.

A trend is observed in favour of the repair group (p=0.050).

These studies exhibit none heterogeneity for this outcome measure (I²=0%).

**
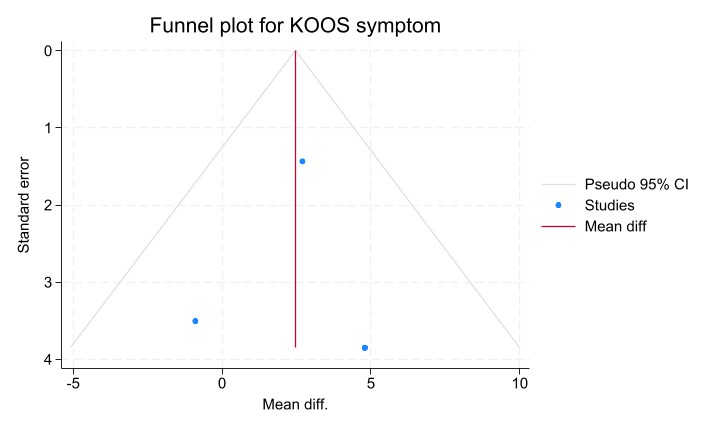
**

***Funnelplot_MA_KOOS_symptom.pdf***

The funnel plot for the KOOS symptom outcome does not reveal any evidence of publication bias.

**KOOS activities of daily**


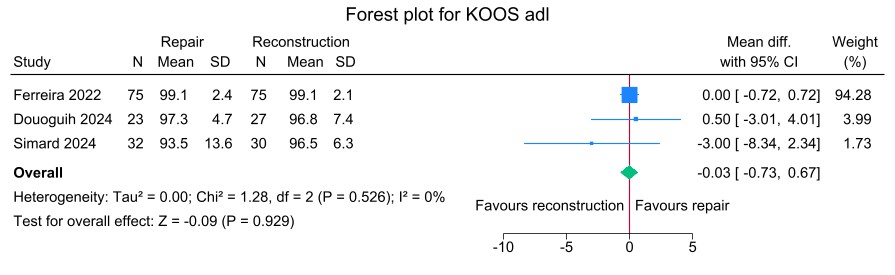


***Forestplot_MA_KOOS_adl.pdf***

There is no significant difference between the repair group and the reconstruction group for the KOOS adl outcome (p=0.929).

These studies exhibit none heterogeneity for this outcome measure (I²=0%).

**
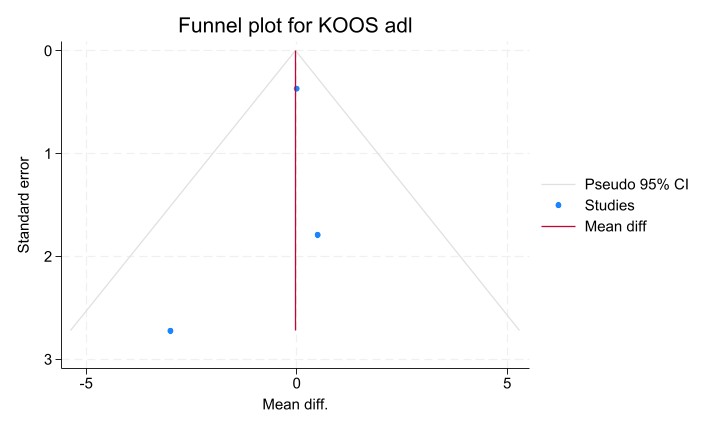
**

***Funnelplot_MA_KOOS_adl.pdf***

The funnel plot for the KOOS adl outcome does not reveal any evidence of publication bias.

**KOOS sport**


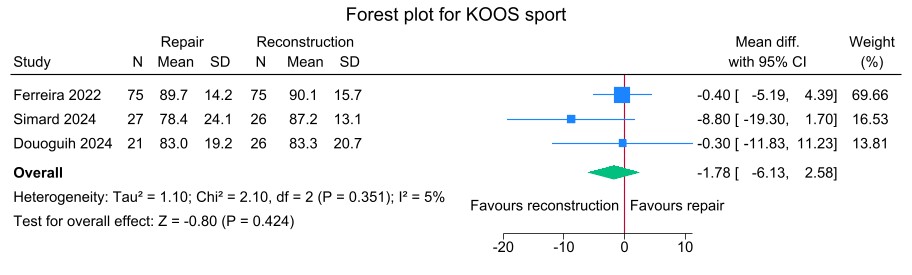


***Forestplot_MA_KOOS_sport.pdf***

There is no significant difference between the repair group and the reconstruction group for the KOOS sport outcome (p=0.424).

These studies exhibit low heterogeneity for this outcome measure (I²=5%).

**
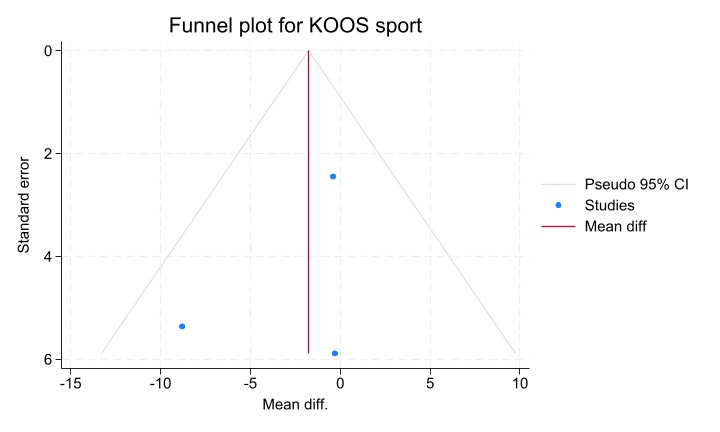
**

***Funnelplot_MA_KOOS_sport.pdf***

The funnel plot for the KOOS sport outcome does not reveal any evidence of publication bias.

**KOOS quality of life**


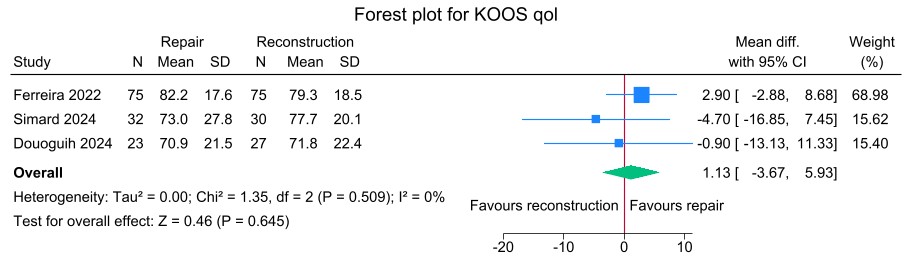


***Forestplot_MA_KOOS_qol.pdf***

There is no significant difference between the repair group and the reconstruction group for the KOOS qol outcome (p=0.645).

These studies exhibit none heterogeneity for this outcome measure (I²=0%).

**
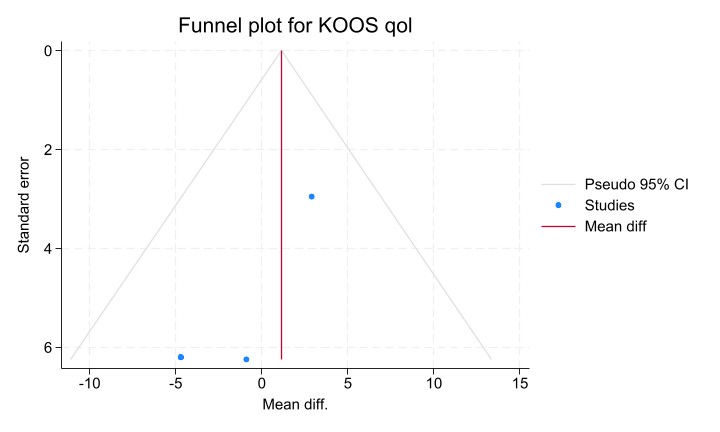
**

***Funnelplot_MA_KOOS_qol.pdf***

The funnel plot for the KOOS qol outcome does not reveal any evidence of publication bias.

**VAS pain**


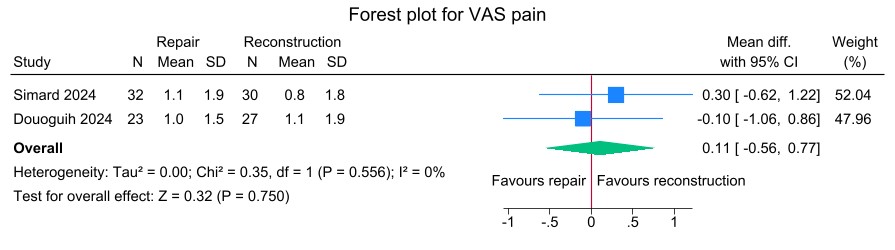


***Forestplot_MA_VAS_pain.pdf***

There is no significant difference between the repair group and the reconstruction group for the VAS pain outcome (p=0.750).

These studies exhibit none heterogeneity for this outcome measure (I²=0%).

**
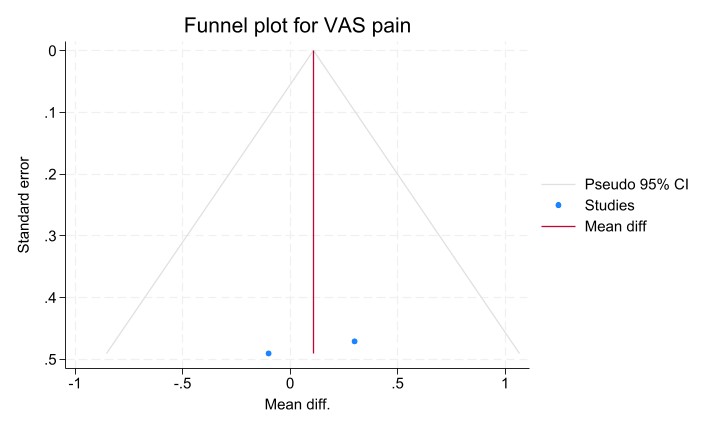
**

***Funnelplot_MA_VAS_pain.pdf***

The funnel plot for the VAS pain outcome does not reveal any evidence of publication bias.

**SANE**


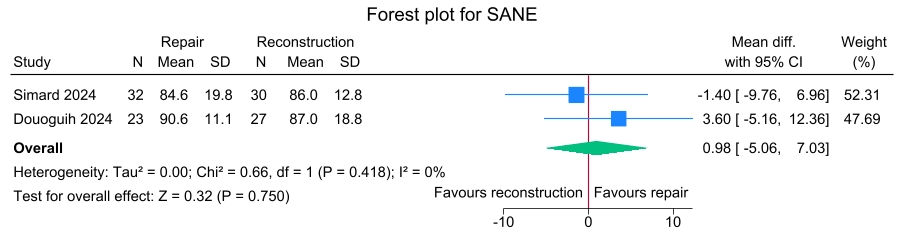


***Forestplot_MA_SANE.pdf***

There is no significant difference between the repair group and the reconstruction group for the SANE outcome (p=0.750).

These studies exhibit none heterogeneity for this outcome measure (I²=0%).

**
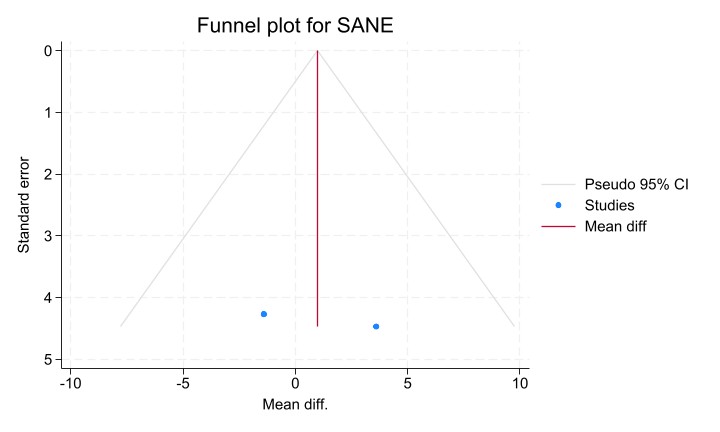
**

***Funnelplot_MA_SANE.pdf***

The funnel plot for the SANE outcome does not reveal any evidence of publication bias.

**Tegner**


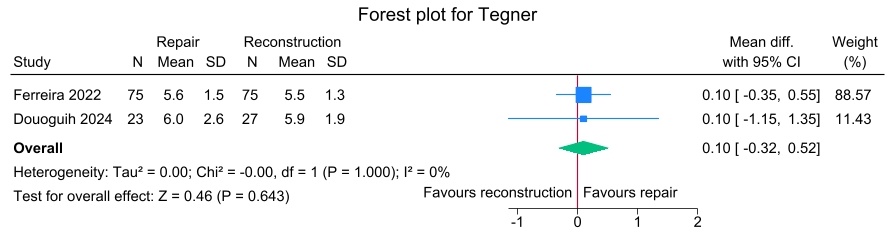


***Forestplot_MA_Tegner.pdf***

There is no significant difference between the repair group and the reconstruction group for the Tegner outcome (p=0.643).

These studies exhibit none heterogeneity for this outcome measure (I²=0%).

**
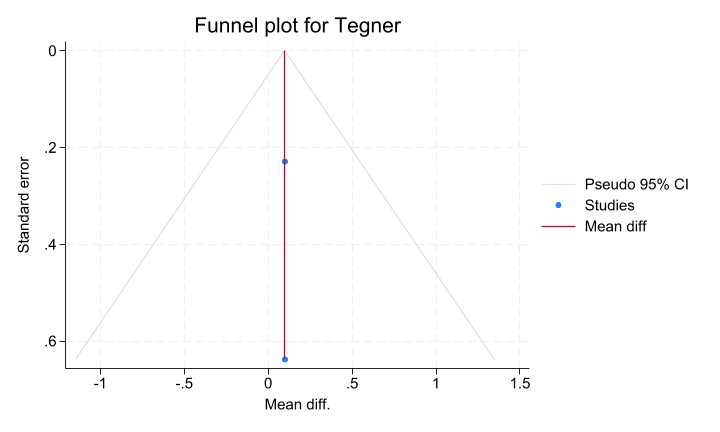
**

***Funnelplot_MA_Tegner.pdf***

The funnel plot for the Tegner outcome does not reveal any evidence of publication bias.

**Lysholm**


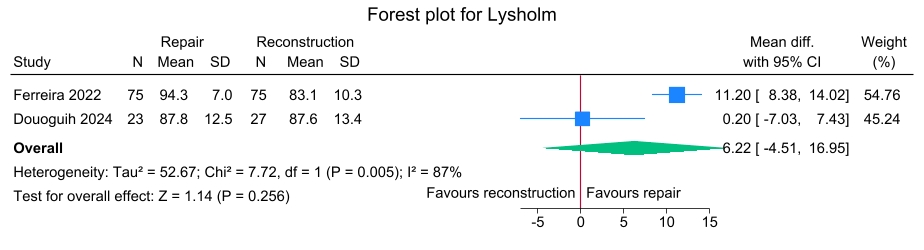


***Forestplot_MA_Lysholm.pdf***

There is no significant difference between the repair group and the reconstruction group for the Lysholm outcome (p=0.425).

These studies exhibit none heterogeneity for this outcome measure (I²=0%).

**
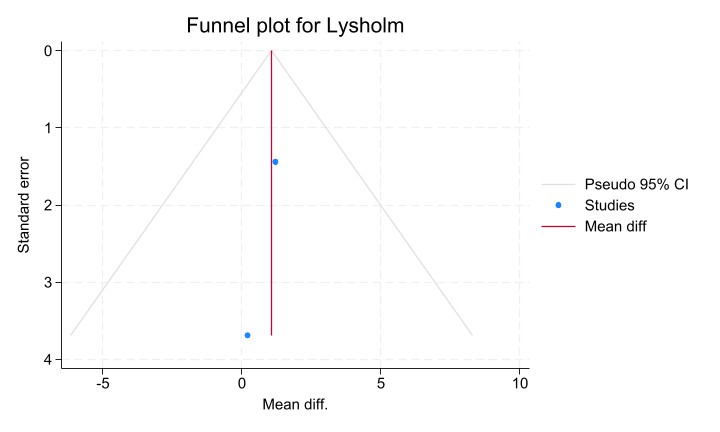
**

***Funnelplot_MA_Lysholm.pdf***

One of the two studies fall outside the expected boundaries in the funnel plot for the Lysholm outcome, showing notable dispersion. Given the small number of studies (n=2), conclusions regarding publication bias are limited. The observed dispersion may also indicate potential heterogeneity between studies, necessitating caution in interpretation.

**KT1000**


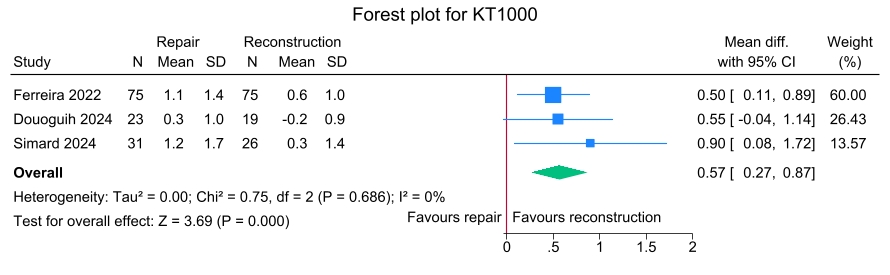


***Forestplot_MA_KT1000.pdf***

There is a significant difference between the repair group and the reconstruction group for the KT1000 outcome. The mean difference in KT1000 is 0.57 [95% CI: 0.27, 0.87], favouring the reconstruction group.

**
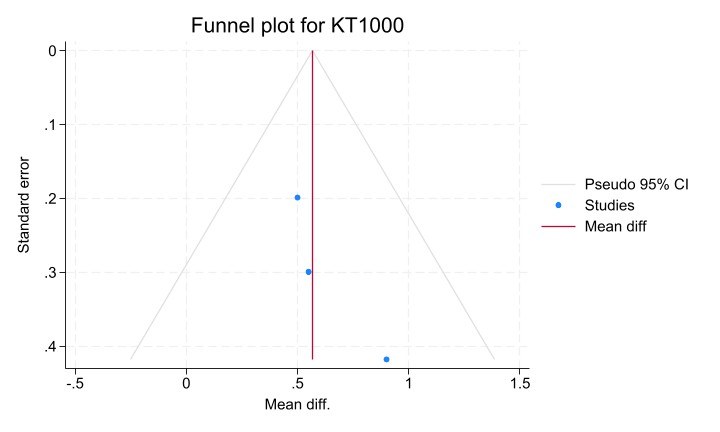
**

***Funnelplot_MA_KT1000.pdf***

The funnel plot for the KT1000 outcome does not reveal any evidence of publication bias.
